# Transformation from calcium sulfate to calcium phosphate in biological environment

**DOI:** 10.1007/s10856-021-06622-7

**Published:** 2021-12-04

**Authors:** Ying-Cen Chen, Wei-Hsing Tuan, Po-Liang Lai

**Affiliations:** 1 Department of Materials Science and Engineering, National Taiwan University, Taipei, Taiwan; 2 Department of Orthopedic Surgery, Bone and Joint Research Center, Chang Gung Memorial Hospital at Linkou, College of Medicine, Chang Gung University, Taoyuan, Taiwan

## Abstract

The formation of a nano-apatite surface layer is frequently considered a measure of bioactivity, especially for non-phosphate bioceramics. In the present study, strontium-doped calcium sulfate, (Ca,Sr)SO_4_, was used to verify the feasibility of this measure. The (Ca,Sr)SO_4_ specimen was prepared by mixing 10% SrSO_4_ by weight with 90% CaSO_4_·½H_2_O powder by weight. A solid solution of (Ca,7.6%Sr)SO_4_ was then produced by heating the powder mixture at 1100 °C for 1 h. The resulting (Ca,Sr)SO_4_ specimen was readily degradable in phosphate solution. A newly formed surface layer in the form of flakes was formed within one day of specimen immersion in phosphate solution. Structural and microstructure–compositional analyses indicated that the flakes were composed of octacalcium phosphate (OCP) crystals. An amorphous interface containing OCP nanocrystals was found between the newly formed surface layer and the remaining (Ca,Sr)SO_4_ specimen. The specimen was also implanted into a rat distal femur bone defect. In addition to new bone, fibrous tissue and inflammatory cells were found to interlace the (Ca,Sr)SO_4_ specimen. The present study indicated that a more comprehensive evaluation is needed to assess the bioactivity of non-phosphate bioceramics.

**Figure:**
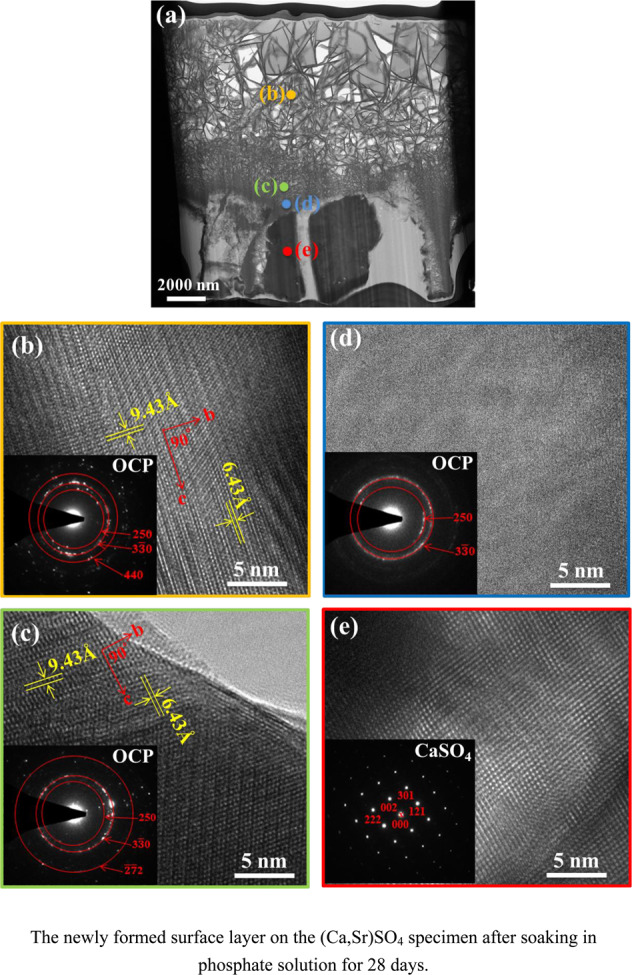
The newly formed surface layer on the (Ca,Sr)SO_4_ specimen after soaking in phosphate solution for 28 days.

The newly formed surface layer on the (Ca,Sr)SO_4_ specimen after soaking in phosphate solution for 28 days.

## Introduction

Though bioactivity is an important issue for bioactive ceramics, it is challenging to define the extent of bioactivity. Since apatite comprises much of the structure and composition of bone, the presence of a nano-apatite surface layer was frequently considered a measure of bioceramic bioactivity [1]. Indeed, the presence of calcium phosphate could induce the differentiation of mesenchymal stem cells to bone tissue [2]. A recent study further indicated that the attachment of cells could be related to the re-structuring of protein on apatite [3]. Thamma et al. suggested that the bioactivity of the bioceramic was determined only by its surface, with the composition of the bulk bioceramic itself mattering little [3].

An interesting example of bioceramics is bioactive glass [4]. The bioactive glass is usually composed of silicate units, SiO_4_^4−^, and calcium ions, Ca^2+^. During the soaking of the bioactive glass in simulated body fluid, a carbonated hydroxyapatite (CHA) surface layer was observed within a matter of minutes [5]. The size of the apatite crystals in this CHA layer was on the nanoscopic scale. The formation of such a nano-apatite layer was believed to be the key to the bioactivity of bioactive glass [6, 7].

In the present study, such a hypothesis is evaluated with a non-phosphate bioceramic, the solid solution of calcium sulfate and strontium sulfate, (Ca,Sr)SO_4_. This solid solution had been implanted into rats, and new bone had been observed at the interface between implant and bone [8]. However, the bioactivity of this solid solution had not been investigated.

Calcium sulfate exists in three levels of hydration, calcium sulfate dihydrate (CaSO_4_·2H_2_O), calcium sulfate hemihydrate (CaSO_4_·½H_2_O) and calcium sulfate anhydrite (CaSO_4_). The hemihydrate is unique in its self-setting capability, namely, it transforms to the dihydrate through the addition of water [9]. Furthermore, this transformation is accompanied with the release of a small amount of heat. The hemihydrate had thus been used either as the outside fixture for bone fracture or the inner filler for bone defect for more than a hundred years [10]. Apart from the hemihydrate, recent studies had demonstrated that the anhydrite could also be used as a filler for bone defects [8]. The anhydrite could be prepared by heating the hemihydrate or the dihydrate above 220 °C [9, 11]. During heat treatment at elevated temperatures, some useful ions, such as silicon [12] or strontium [13], could be dissolved into the anhydrite phase. For example, the addition of strontium sulfate into calcium sulfate hemihydrate could produce a solid solution of strontium-doped calcium sulfate, (Ca,Sr)SO_4_, after heating above 1000 °C. The Sr ions could enhance bone formation and delay bone resorption [14]. Furthermore, the addition of Sr ions barely affected the degradation of CaSO_4_ [8]. Regardless, this solid solution is not a phosphate, and the mechanism for its bioactivity has not been addressed. In the present study, the degradation behavior of a (Ca,Sr)SO_4_ solid solution both in vitro and in vivo is investigated. Further attention is given to the surface layer of the solid solution. The relationship between the surface apatite layer and its bioactivity is investigated.

## Experimental procedures

### Specimen preparation and its characterization

Two powders, calcium sulfate hemihydrate (CaSO_4_·1/2H_2_O, JT Baker, USA) and strontium sulfate (SrSO_4_, Alfa Aesar, USA) powders, were used as the starting materials. The weight fraction of SrSO_4_ in the powder mixture was 10%. Two powders were mixed together in a turbo mixer with milling media of zirconia balls and ethyl alcohol. The milling time was 4 h. After drying in a rotary evaporator, the dried powder lumps were crushed with mortar and pestle. Disc specimens were prepared by die-pressing at 25 MPa. A heat treatment was carried out at 1100 °C for 1 h. Since the solubility of strontium sulfate into calcium sulfate was higher than 10 wt% [13], it would result in a (Ca,7.6%Sr)SO_4_ solid solution.

The density of the specimen was assessed through the measurement of dimensions and weight. The microstructure of the specimen was characterized using two techniques, scanning electron microscopy (SEM, JSA6510, JEOL, Japan) and transmission electron microscopy (TEM, Talos F200X, Thermo Fisher Scientific, USA). For the SEM observation, both the surface and cross section of the degraded specimens were observed. The polished section of the specimen was also observed. To estimate the grain size, a thermal etching at 1000 °C for 1 h was used to reveal the grain boundaries. A line intercept technique was applied to determine the grain size. More than 200 grains were counted. For the TEM characterization, a focus ion beam technique (FIB, Helios NanoLab 400 s, Thermo Fisher Scientific Co., USA) was used to collect a thin section from the specimen. The TEM electron beam was 2 nm; the area for diffraction analysis was about 200 nm. The local composition was analyzed through TEM-EDX (Energy-dispersive X-ray) technique.

The phase analysis was conducted using X-ray diffraction (XRD, TTRAX 3, Rigaku Co., Japan) and Raman spectroscopic techniques (iHR550, Horiba Co., Japan). For Raman technique, Ar^+^ laser beam with wavelength of 514.5 nm was used to excite the specimen. Both the biaxial strength and compressive strength were measured. The compressive strength of specimens was measured using a universal testing machine (MTS 810, MTS Co., USA). The ratio of height to diameter was close to unity. The loading rate was 1 mm/min; five specimens were measured. The flexural strength was measured under a biaxial load. The load was applied through a ball-on-three-balls jig. The diameter of the disc specimens was 21 mm. The displacement rate was 0.48 mm/min; four specimens were used.

### Degradation behavior in phosphate solution

The degradation behavior of (Ca,Sr)SO_4_ specimen was evaluated in a phosphate buffered saline (PBS, Gibco Co. USA) solution. The composition of the solution, as reported by the manufacturer, was potassium chloride (KCl, 200 mg/L), potassium phosphate monobasic (KH_2_PO_4_, 200 mg/L), sodium chloride (NaCl, 8000 mg/L) and sodium phosphate dibasic (Na_2_HPO_4_^.^7H_2_O, 2160 mg/L). The specimen weight to the solution volume was kept at a constant ratio of 1 mg: 10 ml. The specimen and solution were shaken within a test tube to simulate the dynamic situation in vivo. The solution was refreshed every day. After each day, the remain of disc specimen was collected from the solution, then dried in an oven at 100 °C for 1 h. The weight of the specimen was measured. The rate of degradation was expressed in terms of weight loss on a daily basis. The concentration of Ca and Sr ions in the collected solution was measured with an inductively coupled plasma-mass spectroscopy (ICP-MS, Perkin Elmer Co., USA). The [Ca^2+^] and [Sr^2+^] in the fresh PBS were measured with the same technique. The pH value of the solution was also monitored.

### In vivo study

The animal model used in the present study was a rat distal femur model. The study was approved by Chung Gung Memorial hospital (approval number: IACUC 2016092004). Four Sprague Dawley rats were used. Cylindrical defect, with the size of 3 mm in diameter and 4 mm in depth, was introduced into the distal femur by a drill. The (Ca,Sr)SO_4_ specimen of the same size was press fit into the defect. The recovery of these rats after surgery was normal. The rats were sacrificed 3 months postoperatively. The micro computed tomography (micro-CT, NanoSPECT/CT, MediSo Co., Hungary) of the femur was taken first. Before the histology observation, the femur was dehydrated, decalcificated, and then fixed in paraffin, cut into thin slices (thickness: 4-5 um). Masson’s trichrome (ArrayBiotech Co., Taiwan) were used as the stain.

## Results

### Characteristics of the specimens

**Fig. 1:**
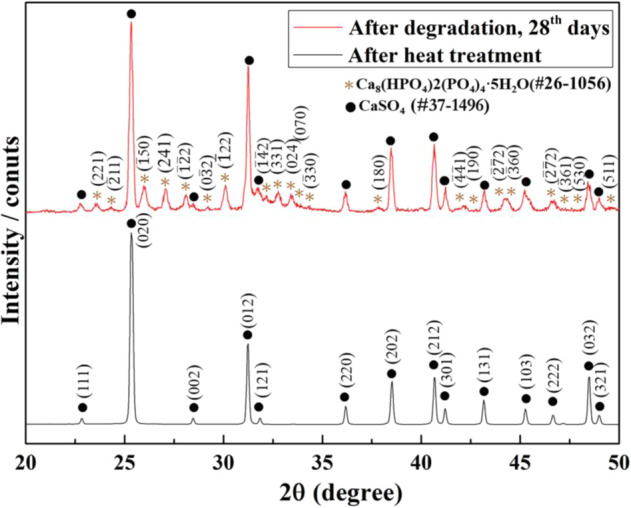
XRD patterns of the (Ca,Sr)SO_4_ specimen after heat treatment (lower pattern) and after degradation in phosphate solution for 28 days (upper pattern).

The calcium sulfate hemihydrate releases its water of crystallization at a temperature above 220 °C [9, 11]. The hemihydrate thus transforms to the anhydrite before the heat-treatment temperature, 1100 °C, is reached. The starting composition of the specimen is 90% CaSO_4_·½H_2_O and 10% SrSO_4_ by weight. After heating at 1100 °C for 1 h, only the anhydrite is detected in the sintered specimen (Fig. 1). This indicates that the SrSO_4_ dissolved into the CaSO_4_ to form (Ca,Sr)SO_4_ at the elevated temperature. The resulting composition after heat treatment is (Ca,7.6%Sr)SO_4_. Since the theoretical densities of CaSO_4_ and SrSO_4_ are 2.96 g/cm^3^ [15, 16] and 3.96 g/cm^3^ [17], respectively, the theoretical density of (Ca,7.6%Sr)SO_4_ is 3.06 g/cm^3^. The density after heat treatment at 1100 °C for 1 h is 2.84 ± 0.01 g/cm^3^, corresponding to a relative density of 93.5 ± 0.3%. The remaining pores within the specimen are not likely interconnected [18]. This is confirmed by dropping water onto the specimen, where no water penetration is observed. The interactions of the specimen in aqueous media would therefore start from the surface. These interactions include the degradation of (Ca,Sr)SO_4_ in phosphate solution (in vitro), as well as the formation of new bone within bone defects (in vivo). In contrast to porous bone grafts [19], the use of a dense disc specimen allows for unambiguous opportunity to locate the sites where reactions take place.

The grain size, as determined with the line intercept technique, is 20 μm (Table 1). The biaxial strength of the specimen is 29 ± 2 MPa. The compressive strength is 70 ± 18 MPa, which is close to that of cortical bone [20, 21].

**Table 1:** Characteristics of the (Ca,Sr)SO_4_ specimen investigated in the present study.

Characteristics of (Ca,Sr)SO_4_ specimen	Values
SrSO_4_ content in the starting powder mixture/wt%	10
Sr content in (Ca,Sr)SO_4_/mol%	7.6
Density after heat treatment/g/cm^3^	2.84 ± 0.01
Theoretical density/g/cm^3^	3.06
Relative density/%	93.5 ± 0.3
Grain size/μm	20
Strength under biaxial load/MPa	29 ± 2
Strength under compressive load/MPa	70 ± 18

### Degradation of the (Ca,Sr)SO_4_ specimen in phosphate solution

**Fig. 2:**
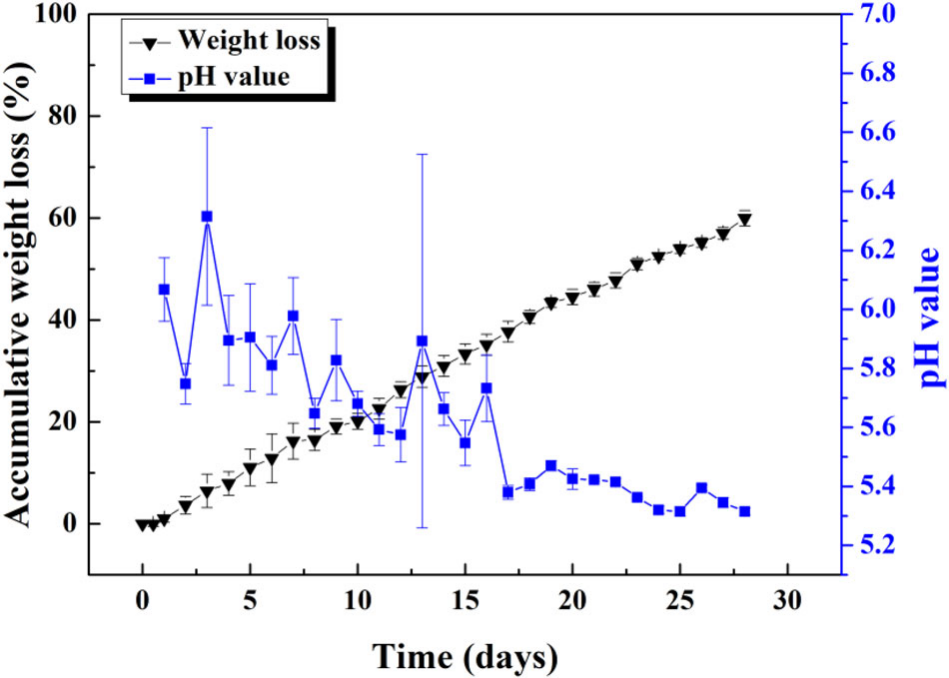
Daily weight loss of the (Ca,Sr)SO_4_ specimen in phosphate solution. The daily pH value of the phosphate solution after soaking of the specimen is also shown. The phosphate solution is refreshed every day.

**Fig. 3:**
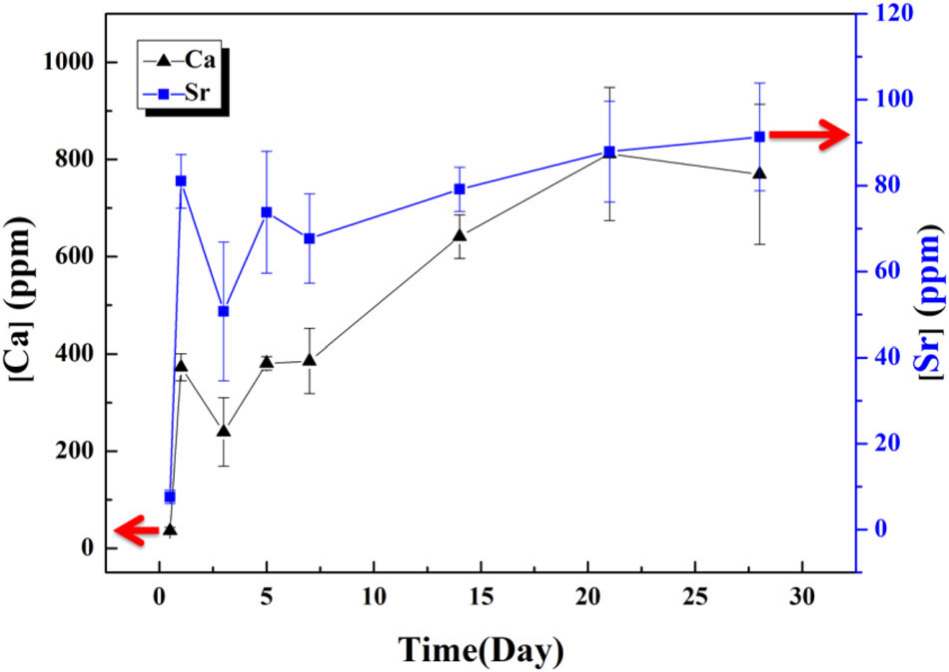
Concentration of Ca and Sr ions in the phosphate solution after soaking the (Ca,Sr)SO_4_ specimen. The phosphate solution is refreshed every day.

The degradation behavior of the (Ca,Sr)SO_4_ specimen is evaluated by soaking it in a phosphate solution. Figure 2 shows the weight loss of the disc specimen in the solution within a time span of 28 days. The phosphate solution was refreshed every 24 h. The weight of the specimen was monitored every day, and the pH value of the solution was also measured daily. The weight loss takes place at a steady rate of ~2.2% per day. From this figure, we can estimate that a complete degradation may take place at roughly 45 days. The pH value of the solution is lower than 7 and decreases slightly with time. The concentration of Ca and Sr ions in the solution is shown in Fig. 3. The [Ca^2+^] in the fresh phosphate solution is very low, at 4.0 ± 0.3 ppm, as determined with the ICP technique. The [Ca^2+^] in the solution after soaking the specimen is much higher than this value. In the first few days, [Ca^2+^] in the solution is ~300 ppm, before gradually increasing to ~600 ppm. There is no [Sr^2+^] in the fresh phosphate solution. After the specimen is soaked, ~80 ppm of Sr ions is released into the solution per day.

**Fig. 4:**
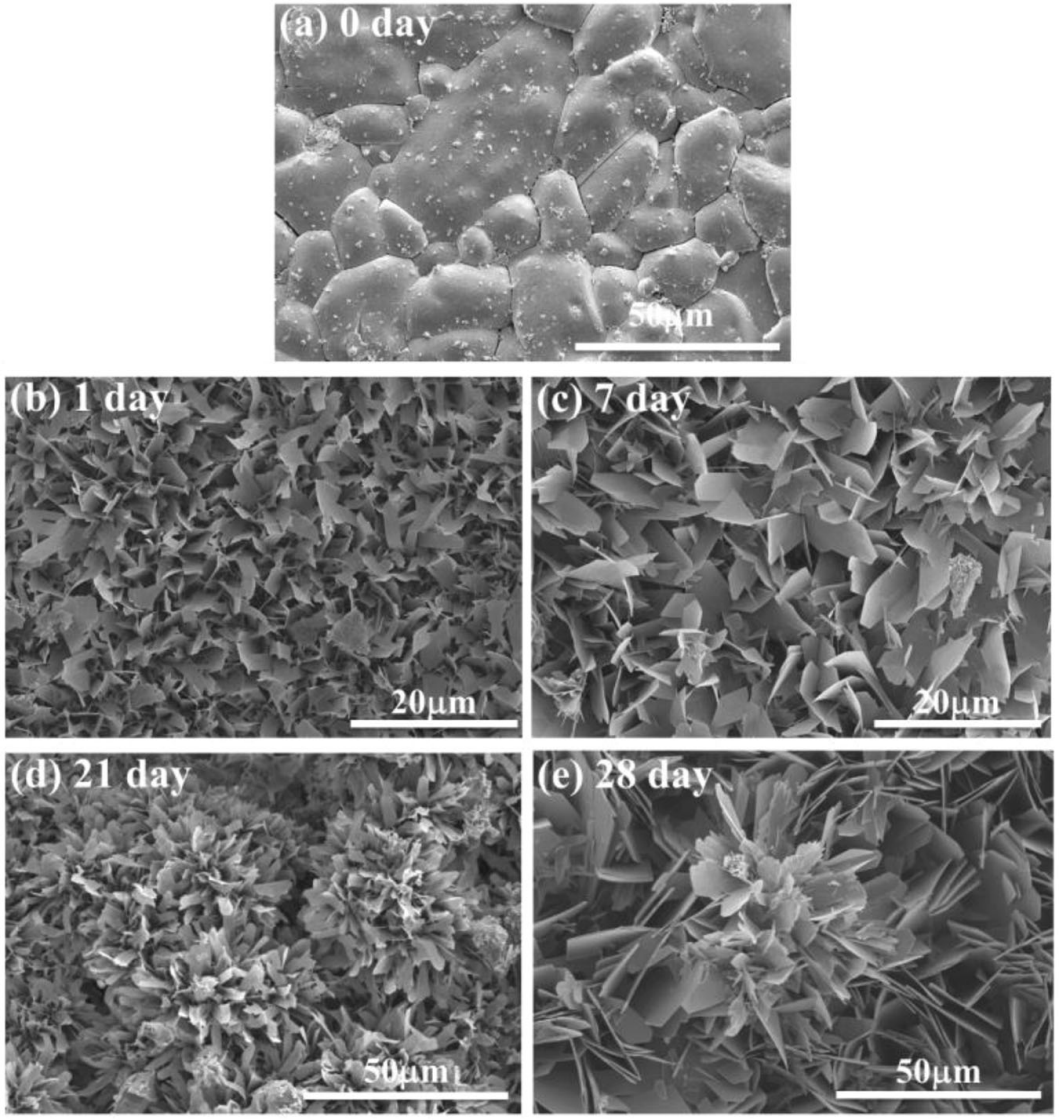
**a** Surface morphology of the (Ca,Sr)SO_4_ specimen before soaking. **b**–**e** Surface morphology of the specimen after soaking in phosphate solution for (**b**) 1, (**c**) 7, (**d**) 21, and (**e**) 28 days. The phosphate solution is refreshed every day.

**Fig. 5:**
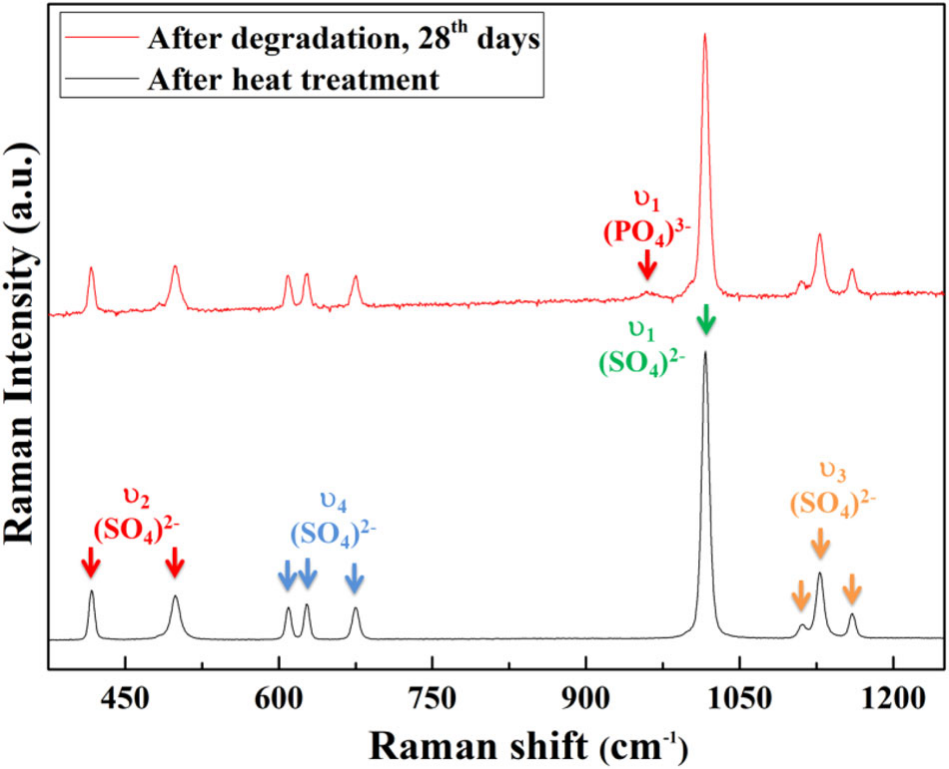
Raman spectroscopy of the (Ca,Sr)SO_4_ specimen after heat treatment (lower spectrum) and after degradation in phosphate solution for 28 days (upper spectrum).

The surface of the disc specimen before degradation is shown in Fig. 4(a). The specimen surface is dense and rather smooth after heat treatment. The SEM micrograph demonstrates that the size of the (Ca,Sr)SO_4_ grains is around 20 μm. After soaking in the phosphate solution for one day, small flakes with a width of 1 to 2 μm and thickness of <0.1 μm are observed (Fig. 4b). The size of the flakes increases with soaking time (Fig. 4c–e). After degradation in phosphate solution, the XRD analysis detects a new phase (Fig. 1) apart from the calcium sulfate anhydrite (PDF #37-1496). The XRD peaks for the new phase are broad and low. These peaks may belong to either hydroxyapatite (HAp, Ca_10_(PO_4_)_6_(OH)_2_; PDF #73-0294) or octacalcium phosphate (OCP, Ca_8_(HPO_4_)_2_(PO_4_)_4_·5H_2_O; PDF #26-1056). Apart from the XRD analysis, Raman spectroscopy was also performed on the specimen after degradation (Fig. 5). The spectrum for the calcium sulfate specimen before degradation is also shown for comparison. The fingerprint for the PO_4_^3−^ of OCP is detected [22, 23]. Both phase analysis techniques, XRD and Raman spectroscopy, confirm the formation of apatite on the surface of the (Ca,Sr)SO_4_ specimen after degradation in phosphate solution. The crystalline structure of the apatite is mainly OCP.

### Transformation from calcium sulfate to calcium phosphate in phosphate solution

**Fig. 6:**
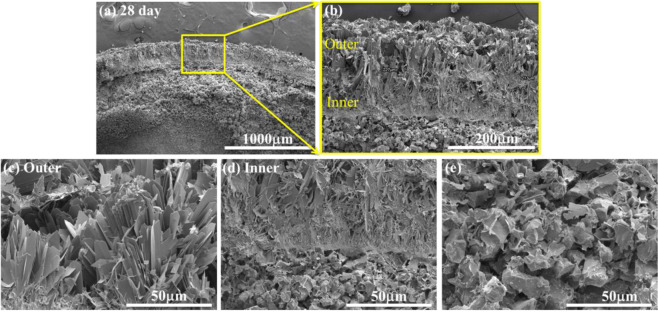
**a** Cross-section of the (Ca,Sr)SO_4_ specimen after soaking in phosphate solution for 28 days. **b** Newly formed surface layer. The (**c**) outer and (**d**) inner parts of the surface layer. **e** The coarse-grain layer under the newly formed surface layer.

Observation of a cross-section of the degraded specimen is then conducted. Figure 6(a) shows a typical cross-section of the (Ca,Sr)SO_4_ specimen after soaking in phosphate solution for 28 days. A newly formed layer, around 200 μm, is observed on the specimen surface (Fig. 6b). The outer part of the newly formed layer is mainly composed of large flakes (Fig. 6c). The flakes reach a length of up to 50 μm; nevertheless, the flakes remain thin (<1 μm) after soaking in phosphate solution for 28 days. The flakes are smaller toward the inner part of the surface layer. The inner part is relatively dense (Fig. 6d). A porous region containing large grains (Fig. 6e) is found under the newly formed layer. This porous region is around 500 μm in thickness (Fig. 6a). Some small flakes are observed on the surface of the large grains (Fig. 6e).

**Fig. 7:**
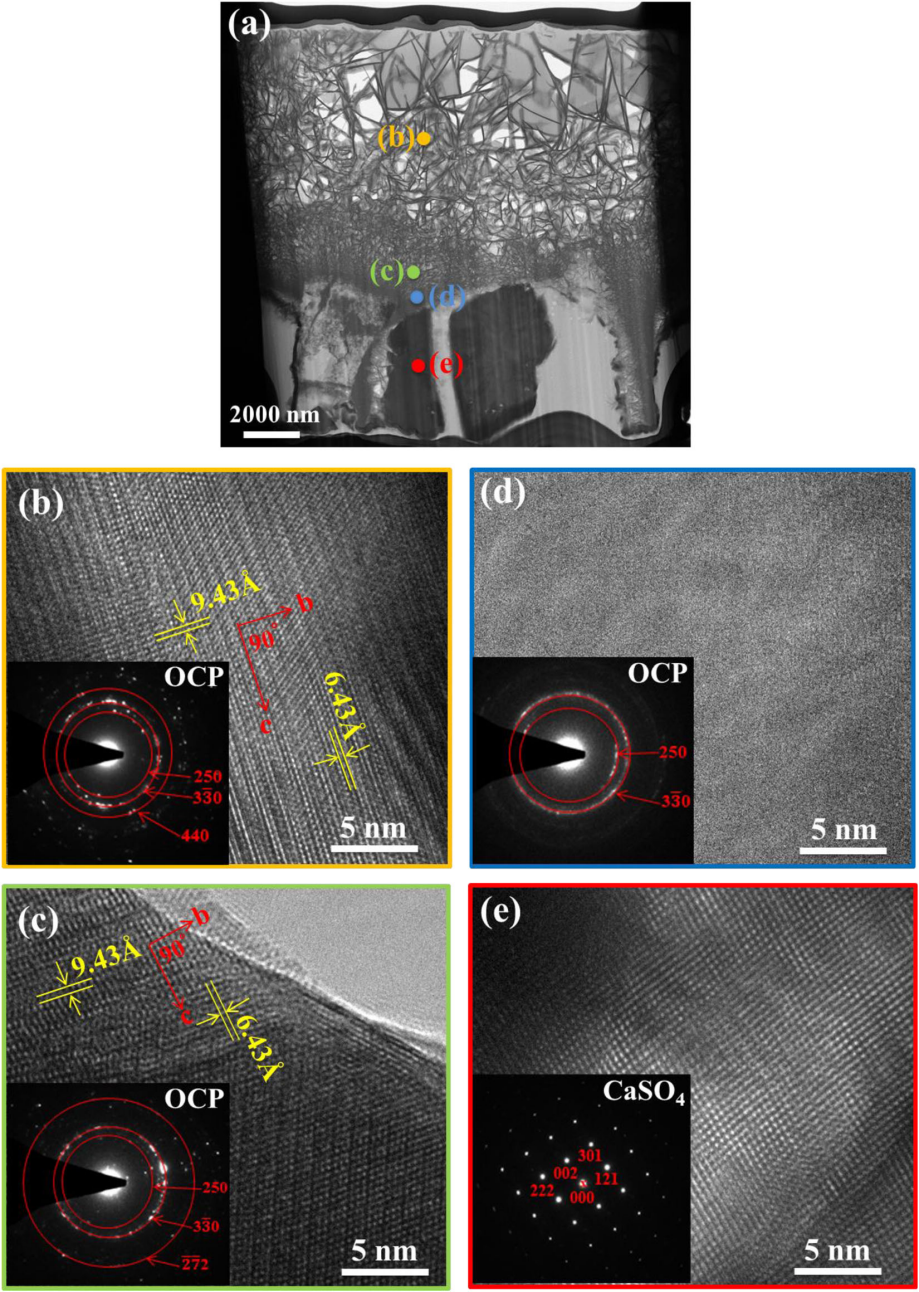
**a** TEM micrograph for the newly formed surface layer on the (Ca,Sr)SO_4_ specimen after soaking in phosphate solution for 28 days. **b**–**e** Corresponding high-resolution TEM micrographs at the locations indicated in (**a**). The diffraction pattern is shown in the inset.

Structural and compositional analyses on the newly formed layer are also conducted using TEM techniques. A low-magnification bright-field image for the newly formed layer is shown in Fig. 7(a). This TEM micrograph confirms that the newly formed surface layer is composed of flakes. The flakes on the outer region are relatively large. A typical lattice image for the large flake is shown in Fig. 7(b). Apart from several diffraction spots, diffraction rings are observed. This suggests that the large flakes are composed of many finer grains. The ring pattern indicates that the crystalline phase of the flake comprises octacalcium phosphate (OCP) [24]. Another high-resolution bright-field image is taken near the bottom of the outer porous layer (Fig. 7c). The flakes in this region are much smaller in size. The ring pattern indicates that many fine OCP crystals are present within these small flakes. Phase analyses using the TEM technique (Fig. 7b, c) confirms the results from the XRD and Raman analyses indicating that the crystalline phase of the newly formed surface layer is OCP. Furthermore, the size of the OCP crystals decreases toward the bottom of this surface layer.

A thin layer is observed at the interface between the surface layer and the large grains (Fig. 7d). This interface is relatively thin, measuring ~300 nm. A high-resolution bright field image is taken at this layer. This high-resolution TEM micrograph depicts an amorphous characteristic; nevertheless, the diffused ring pattern suggests that there are many OCP nanocrystals (<5 nm) within this interface.

The grains under the newly formed surface layer are large, and two such grains are shown in Fig. 7(a). The diffraction pattern of one large grain, shown in the inset, suggests that it is an orthorhombic CaSO_4_ grain [25]. This confirms that this porous layer is a remnant of the specimen after degradation.

The corresponding TEM-EDX analysis (Table 2) confirms the phase analysis results. The composition of the newly formed layer is mainly calcium and phosphorus. The Ca/P ratio is around 1.4, which is a value close to that of OCP (Ca_8_(HPO_4_)_2_(PO_4_)_4_·5H_2_O). Furthermore, the amount of Sr in this calcium phosphate layer is very low. The remaining substance after degradation is calcium sulfate (Fig. 7e). The Ca content of the large grain is relatively low; instead, the Sr content is high. The interface, shown in Fig. 7(d), is a transition region between sulfate and phosphate, containing Ca^2+^, Sr^2+^, PO_4_^3−^, and SO_4_^2−^ ions (Table 2).

**Table 2:** The TEM-EDX results for the micrographs shown in Fig. 7b–e.

Location	Ca/at%	Sr/at%	O/at%	P/at%	S/at%
Fig. 7(b)	23.3 ± 5.6	0.7 ± 0.4	58.9 ± 2.4	16.5 ± 4.2	0.6 ± 0.2
Fig. 7(c)	25.4 ± 6.0	0.8 ± 0.4	55.7 ± 2.4	17.5 ± 4.3	0.6 ± 0.2
Fig. 7(d)	8.7 ± 1.4	13.5 ± 2.1	57.0 ± 5.3	6.0 ± 1.3	14.9 ± 3.1
Fig. 7(e)	2.0 ± 0.4	19.2 ± 7.4	56.5 ± 2.0	1.4 ± 0.3	20.9 ± 4.0

### In vivo testing

**Fig. 8:**
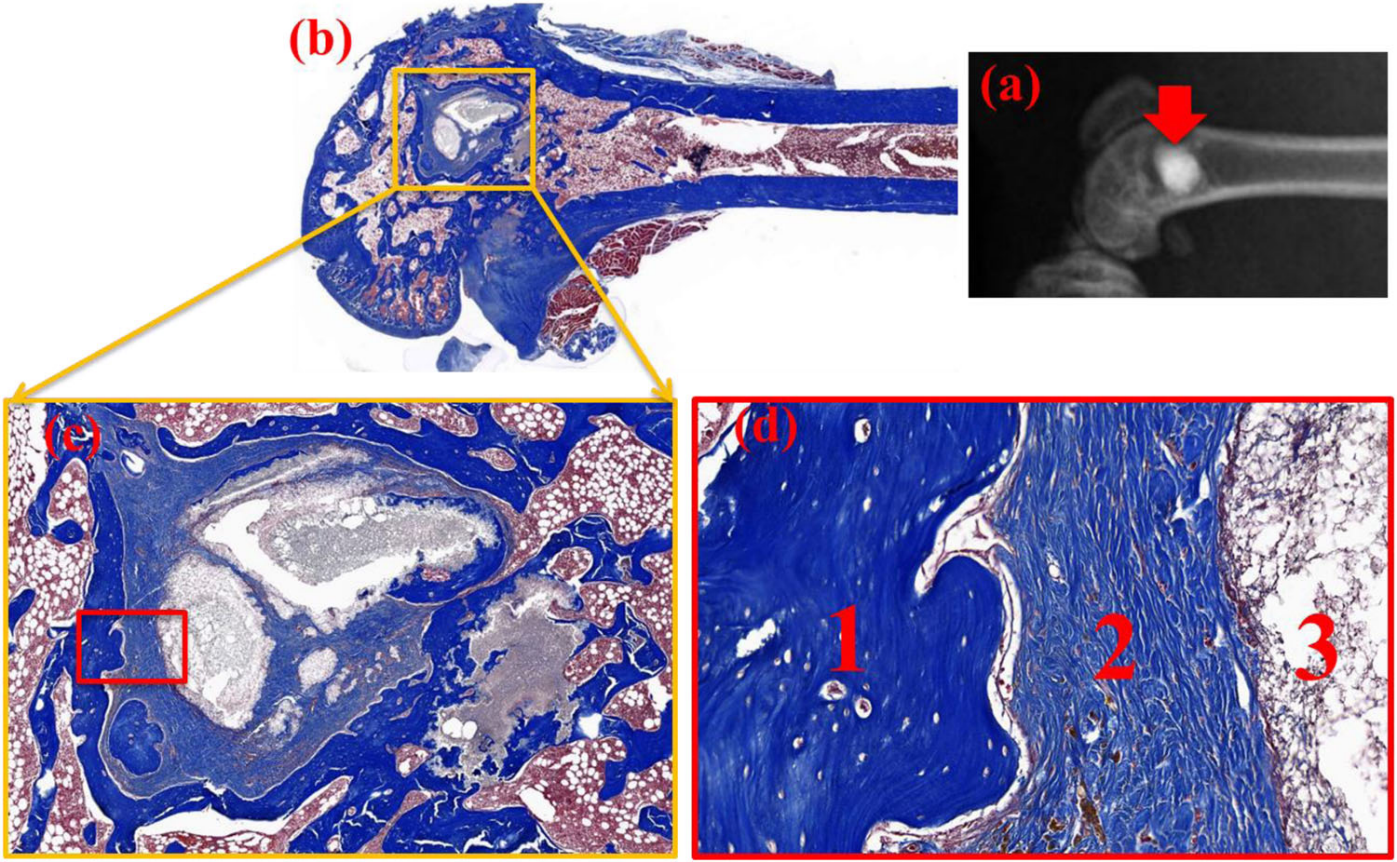
**a** A 3-month post-operative micro-CT image of the bone defect on the rat distal femur. The remaining (Ca,Sr)SO_4_ specimen is indicated by an arrow. The corresponding histology after decalcification is shown in (**b**–**d**). The histology at (**b**) low, (**c**) intermediate, and (**d**) high magnifications. In (**d**), region 1 corresponds to mineralized bone matrix (new bone), with some osteocytes present inside the lacune; region 2 corresponds to aligned fibrous tissue, where many arterioles are observed; and region 3 corresponds to a transition zone between the specimen and fibrous tissue. In this region, fibrous cells and inflammatory cells interlace the surface of the disc.

Since the (Ca,Sr)SO_4_ specimen is relatively dense, all of the interactions with the surrounding bone tissue originate from the surface. The three-month post-operative micro-CT image of the rat distal femur is shown in Fig. 8(a). The remaining implant is indicated with an arrow. The (Ca,Sr)SO_4_ implant is no longer one cylindrical disc. The implant has broken into two large pieces and several smaller pieces (Fig. 8b). This indicates that the (Ca,Sr)SO_4_ specimen has degraded in vivo. New bone together with fibrous tissue can be observed (Fig. 8c). Three regions can be defined between the remaining implant (which has since decalcified) and the bone tissue (Fig. 8d). Within the new bone (region 1), some lacunae are observed. Osteocytes are found inside the lacunae. A small gap between region 1 and region 2 is noted; this may have resulted from the dehydration pre-treatment. The location of the (Ca,Sr)SO_4_ implant is surrounded by fibrous tissue (region 2). This fibrous tissue is also found between the broken pieces of the implant. Abundant small arterioles are noted within this region. A transition zone (region 3) is observed between the remaining (Ca,Sr)SO_4_ implant and the fibrous tissue. In this transition zone, fibrous cells and inflammatory cells are observed to interlace the location of implant.

## Discussion

### Transformation from calcium sulfate to calcium phosphate

The chemistry is one of the key factors that can affect the bioactivity of this bone graft. Since the structure and chemistry of human bone mainly comprises calcium phosphates, the formation of apatite is a must for a successful bone graft [26], especially in non-phosphate bioceramics. For example, the formation of an apatite surface layer on bioactive glass is taken as evidence for its bioactivity. Indeed, the presence of an apatite layer induces the attachment, proliferation, and differentiation of cells [3, 27]. In the present study, a non-phosphate bioceramic, CaSO_4_, is used. The transformation from calcium sulfate to calcium phosphate in vitro and in vivo is investigated.

Calcium sulfate is commonly known for its inability to induce osteoinduction and osteogenesis [28]. A small amount of strontium sulfate is thus added to the calcium sulfate. Strontium may enhance bone formation and inhibit bone resorption [14]. Furthermore, the charge of Sr ions is the same as that of Ca ions, and the size of Sr ions is close to that of Ca ions. Therefore, the Sr ions can replace the Ca ions during heat treatment at elevated temperatures [13]. In the present study, Sr replaces 7.6% of the Ca (by mole) in the CaSO_4_. After heating to 1100 °C, apart from the solution of Sr, most of the pores are removed from the (Ca,Sr)SO_4_ specimen. The residual pores are no longer interconnected. The degradation process would therefore start from the surface of the specimen.

**Fig. 9:**
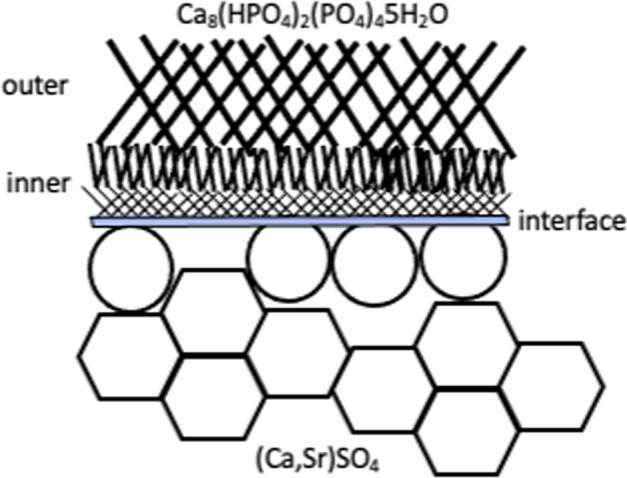
Schematic for the newly formed surface layer on the (Ca,Sr)SO_4_ specimen. The outer surface layer is composed of large Ca_8_(HPO_4_)_2_(PO_4_)_4_·5H_2_O (OCP) flakes. The OCP flakes decrease in size, moving toward the inner surface layer. A thin layer composed of amorphous and OCP nanocrystals is found at the interface between the newly formed surface layer and the remaining (Ca,Sr)SO_4_ specimen. The remaining (Ca,Sr)SO_4_ specimen is no longer dense, demonstrating degradation of the specimen.

A newly formed layer materialized on the surface of the (Ca,Sr)SO_4_ specimen within one day of soaking in PBS (Fig. 4b). Microstructure analysis indicates that the surface layer is composed of nano-crystallized apatite. Structural analyses (XRD and Raman) suggest that the apatite layer is composed of octacalcium phosphate (OCP) crystals. These crystals take the form of flakes. The outer surface is observed to be relatively porous, while the inner layer is observed to be relatively dense (Figs. 6, 7). Furthermore, an amorphous layer is found to be located between the (Ca,Sr)SO_4_ grains and the OCP flakes (Fig. 7d). The TEM-EDX analysis detects Ca^2+^, Sr^2+^, PO_4_^3−^, and SO_4_^2−^ ions in this interface (Table 2). A schematic for the transformation from calcium sulfate to calcium phosphate is shown in Fig. 9. The newly formed surface layer is composed of OCP flakes. The flakes decrease in their size toward the inner part of the layer. Many small flakes are found in the inner part of the surface layer; furthermore, the size of these flakes is smaller than the size of the (Ca,Sr)SO_4_ grains. This implies that a transformation from calcium sulfate to calcium phosphate is likely a multi-step process, similar to the transformation of calcium sulfate from the hemihydrate form to the dihydrate form [29]. The thin interface provides a mass transport path between sulfate and phosphate. Since the weight loss shows a linear relationship with time (Fig. 2), this suggests that the degradation process is a reaction-controlled process. Furthermore, the thin interface is likely to be porous in nature.

The reaction on the surface of the (Ca,Sr)SO_4_ specimen is likely one of dissolution and precipitation. The (Ca,Sr)SO_4_ specimen releases both Ca and Sr ions into solution during degradation (Fig. 3). However, the precipitation process involves the formation of OCP flakes only, and Sr ions remain in solution. The following dissolution and precipitation reactions are likely to have taken place during the degradation process:

Dissolution of strontium-doped calcium sulfate:

1 $$\left( {Ca,Sr} \right)SO_4 = Ca^{2 + } + Sr^{2 + } + SO_4^{2 - }$$

Precipitation of octacalcium phosphate in phosphate solution:

2 $$\begin{array}{l}8\,Ca^{2 + } + 2\left( {HPO_4} \right)^{2 - } + 4\left( {PO_4} \right)^{3 - }\\ \qquad \quad \,+ \,5\,H_2O = Ca_8\left( {HPO_4} \right)_2\left( {PO_4} \right)_4 \cdot 5\,H_2O\end{array}$$

Since the dissolution and precipitation processes take place at a relatively low temperature (37 °C), the OCP crystals that formed are relatively small. The dissolution of Ca from (Ca,Sr)SO_4_ is faster than the dissolution of Sr from (Ca,Sr)SO_4_; therefore, many Sr ions are left behind within the (Ca,Sr)SO_4_ grain (Table 2).

Through the dissolution and precipitation processes described above, the calcium sulfate in the (Ca,Sr)SO_4_ specimen transforms to calcium phosphate. The present study is the first investigation to provide microstructure evidence on the transformation from calcium sulfate to calcium phosphate.

### Bioactivity of (Ca,Sr)SO_4_

The present study conducts both an in vitro degradation test and an in vivo evaluation. The implantation of (Ca,Sr)SO_4_ induces the formation of new bone (Fig. 8), and fibrous tissue and giant cells surround the degradable implant 3 months post-operation (Fig. 8d). Though fibrous tissue and inflammatory cells are necessary for the healing processes, they should disappear sooner rather than later.

The formation of a nano-apatite layer is frequently considered a sign of bioactivity [26, 30]. With the help of such an apatite interface, a bioactive ceramic can form strong bonds with bone tissue. Calcium sulfate has been generally regarded as bio-compatible [10, 28, 31, 32]. However, many previous studies have also indicated that calcium sulfate lacks osteoinductivity [10, 32–36]. The present study demonstrates two important characteristics that support the bioactivity of a (Ca,Sr)SO_4_ specimen. The first characteristic is the formation of a nano-apatite layer (Fig. 7), and the second characteristic is the formation of new bone (Fig. 8). These two pieces of evidence indicate that (Ca,Sr)SO_4_ is not only osteoconductive but also osteoinductive. One of the issues, however, is that the degradation rate of (Ca,Sr)SO_4_ specimen is too high; the degradation rate of the (Ca,Sr)SO_4_ is higher than the rate of new bone formation.

The (Ca,Sr)SO_4_ specimen is degradable both in vitro and in vivo; the specimen is broken into small pieces after degradation. A gap is formed between two broken pieces of the specimen because of degradation of the implant (Fig. 8). Only fibrous tissue is observed within this gap; this demonstrates that the fibrous tissue forms rapidly during the absorption process. At the same time, giant cells are generated during this stage of the healing process (Fig. 8d). This implies that the properties of osteoconductivity and osteoinduction are not sufficient for a resorbable bioceramic; that is, a third characteristic, a degradation rate slower than the formation rate of new bone, is also necessary. Otherwise, any gap that is formed only encourages the formation of fibrous tissue. These findings thus indicate that the degradation rate of the (Ca,Sr)SO_4_ specimen must be slowed down. In the present study, a heat-treatment technique is applied to remove interconnected pores. However, the degradation rate is not low enough. Other possible strategies for decreasing the degradation rate should be considered.

## Conclusions

In the present study, the transformation from strontium-doped calcium sulfate to calcium phosphate in phosphate solution is investigated. Apart from the kinetics of the degradation behavior, the structure and composition of the newly formed calcium phosphate is characterized. Several key findings are listed below:

1. The formation of calcium phosphate on the surface of strontium-doped calcium sulfate is a fast process. This can be related to the fact that the degradation rate of the (Ca,7.6%Sr)SO_4_ specimen is fast, at ~2.2% per day.
2. The newly formed apatite layer is composed of OCP nanocrystals.
3. Both Ca and Sr ions are released from the (Ca,Sr)SO_4_ specimen; however, Sr ions are not precipitated. The amount of Sr in the newly formed calcium phosphate is very low.
4. A rat distal-femur model detects the formation of new bone. However, fibrous cells and inflammatory cells space the gap between new bone and the remaining (Ca,Sr)SO_4_ implant.
5. The present study indicates that calcium sulfate is both osteoconductive and osteoinductive. Nevertheless, the bioactivity of bioceramics is a complicated issue. The formation of a nano-sized apatite surface layer does not guarantee osteogenesis. The degradation rate is another factor that must also be considered.
